# Supplementary material for: Quantifying the Impact of Chronic Obstructive Sialadenitis on Quality of Life
Source: J Clin Med. 2025 Oct 24;14(21):7560. doi: 10.3390/jcm14217560 (PMC12608179; doi:10.3390/jcm14217560)
Supplement: Supplementary file 1 [file jcm-14-07560-s001.zip › Supplementary Material 6.pdf]

Supplementary Material 6. COSQ Score (median and CI) comparison across epidemiologic, clinic and obstructive factors in single- and multi-gland, and global study cases.

**Variable**		**Uniglandular cases (215 glands, n=215)**		**Multiglandular cases (129 glands, n=63)**		**Global study (uni- and multiglandular cases)**	
		**COSQ**	**p value**	**COSQ**	**p value**	**COSQ**	**p value**
**Sex**	*Male*	20.5 (15.0-31.0)	**0.0052**	20.0 (16.8-35.5)	**0.0001**	20.0 (15.8-31.0)	**0.0000**
	Female	28.0 (18.5-38.0)		38.0 (28.0-47.0)		33.0 (22.0-41.0)	
**Gland involvement**	*Submandibular*	25.0 (16.0-35.5)	0.3348	26.0 (19.0-38.0)	**0.0000**	26.0 (16.8-36.2)	**0.0000**
	Parotid	27.0 (20.0-37.0)		40.0 (32.5-49.0)		35.0 (22.8-42.2)	
**Obstructive cause**	*Stenosis*	28.0 (20.0-38.0)	**0.0176**	37.5 (26.8-48.0)	**0.0045**	33.0 (22.0-41.2)	**0.0000**
	Stenosis + LPD	32.0 (28.0-37.5)		55.0 (49.0-55.0)		37.5 (31.0-50.5)	
	Stenosis + Lithiasis	25.0 (22.0-36.0)		-		25.0 (22.0-36.0)	
	LPD	32.0 (24.0-40.0)		28.0 (19.0-44.0)		31 (20.0-41.8)	
	Mucous plug	21.0 (16.5-35.5)		40.0 (25.5-42.5)		28.5 (18.8-41.8)	
	Lithiasis	20.5 (15.0-31.5)		37.0 (22.0-38.0)		22.0 (15.0-35.0)	
**Stenosis grade**	*Combined*	27.5 (9.8-36.8)	0.0565	40.0 (36.8-45.2)	0.2699	31.5 (18.0-39.8)	**0.0043**
	S1	17.0 (15.0-19.0)		-		17.0 (15.0-19.0)	
	S2	28.5 (20.0-39.8)		36.0 (25.0-48.2)		33.5 (21.0-40.8)	
	S3	24.0 (21.2-29.5)		37.0 (26.0-47.5)		29.0 (22.0-37.8)	
	S4	37.0 (36.0-43.0)		42.5 (32.5-56.8)		40.0 (34.0-56.0)	
