# Supplementary material for: Quantifying the Impact of Chronic Obstructive Sialadenitis on Quality of Life
Source: J Clin Med. 2025 Oct 24;14(21):7560. doi: 10.3390/jcm14217560 (PMC12608179; doi:10.3390/jcm14217560)
Supplement: Supplementary file 1 [file jcm-14-07560-s001.zip › Supplementary Material 1 - COSQ in spanish.pdf]

Cuestionario sobre Sialoadenitis Obstructiva Crónica (CSOC) [Santillan et al, 2022]

Afectación local

1. En los ULTIMOS 3 MESES, ¿Cuánto le duele la glándula afectada, al tocarla o presionarla?

0	1	2	3	4
Nada	Poco	Algo	Bastante	Mucho

2. En los ULTIMOS 3 MESES, Durante las comidas, ¿con qué frecuencia nota **hinchazón** de la glándula afectada o de la zona cercana a ella?

0	1	2	3	4
Nunca	Raramente	A veces	Muy a menudo	Siempre

3. En los ULTIMOS 3 MESES, Entre las comidas, ¿con qué frecuencia nota **hinchazón** de la glándula afectada o de la zona cercana a ella?

0	1	2	3	4
Nunca	Raramente	A veces	Muy a menudo	Siempre

Afectación funcional

4. En los ULTIMOS 3 MESES, ¿con qué frecuencia nota la boca seca (disminución de la saliva)?

0	1	2	3	4
Nunca	Raramente	A veces	Muy a menudo	Siempre

5. En los ULTIMOS 3 MESES, la **hinchazón** de la glándula, ¿le molesta al abrir la boca, bostezar o masticar?

0	1	2	3	4
Nada	Poco	Algo	Bastante	Mucho

6. En los ULTIMOS 3 MESES, la **hinchazón** de la glándula, ¿le molesta al hablar o tragar?

0	1	2	3	4
Nada	Poco	Algo	Bastante	Mucho

Afectación social

7. En los ULTIMOS 3 MESES, cuando ocurre la **hinchazón** de la glándula, ¿lo nota la gente de su alrededor?

0	1	2	3	4

Nunca	Raramente	A veces	Muy a menudo	Siempre

8. En los ULTIMOS 3 MESES, ¿se avergüenza de ser visto en público cuando sufre estos síntomas?

0	1	2	3	4
Nunca	Raramente	A veces	Muy a menudo	Siempre

9. En los ULTIMOS 3 MESES, ¿ha cambiado sus hábitos alimenticios por sus síntomas, evitando la toma de algún alimento?

0	1	2	3	4
Nada	Poco	Algo	Bastante	Completamente

10. En los ULTIMOS 3 MESES, ¿ha cambiado sus hábitos sociales o familiares por sus síntomas?

0	1	2	3	4
Nada	Poco	Algo	Bastante	Completamente

11. En los ULTIMOS 3 MESES, ¿afectan sus síntomas al sueño o al descanso nocturno?

0	1	2	3	4
Nada	Poco	Algo	Bastante	Mucho

Afectación ocupacional

12. En los ULTIMOS 3 MESES, ¿afectan sus síntomas en el desarrollo de actividades laborales, académicas o recreativas?

0	1	2	3	4
Nada	Poco	Algo	Bastante	Mucho

13. En los ULTIMOS 3 MESES, debido a la inflamación de la glándula salival, ¿se ha visto obligado a faltar a sus actividades laborales, académicas y/o recreativas?

0	1	2	3	4
Nada	Poco	Algo	Bastante	Mucho

Afectación emocional

14. En los ULTIMOS 3 MESES, ¿la hinchazón de su glándula le provoca irritabilidad o mal humor?

0	1	2	3	4

Nunca	Raramente	A veces	Muy a menudo	Siempre

15. En los ULTIMOS 3 MESES, ¿la hinchazón de su glándula le provoca tristeza o desanimo?

0	1	2	3	4
Nada	Poco	Algo	Bastante	Mucho

Afectación global sobre la salud

16. En los ULTIMOS 3 MESES, ¿cuántas veces ha necesitado tomar analgésicos o acudir al servicio de urgencias, debido a la inflamación de su glándula salival?

0	1	2	3	4
Nunca	Muy pocas (≤ 2 veces)	Algunas veces (3-4 veces)	Bastantes veces (5-6 veces)	Muchas veces (≥7 veces)

17. En los ULTIMOS 3 MESES, ¿cómo cree que afecta la inflamación de su glándula salival en su salud?

0	1	2	3	4
Nada	Poco	Algo	Bastante	Mucho

18. En los ULTIMOS 3 MESES, teniendo en cuenta la inflamación de su glándula salival, ¿cómo considera que es su salud en general?

0	1	2	3	4
Excelente	Buena	Normal	Mala	Muy mala

Muchas gracias por su colaboración

Extraído de Santillán Coello JM et al. Validation of a Spanish chronic obstructive sialadenitis quality of life questionnaire (CSOC).
Acta Otorrinolaringol Esp (Engl Ed). 2023 Mar-Apr;74(2):116-123.
