# Supplementary material for: Quantifying the Impact of Chronic Obstructive Sialadenitis on Quality of Life
Source: J Clin Med. 2025 Oct 24;14(21):7560. doi: 10.3390/jcm14217560 (PMC12608179; doi:10.3390/jcm14217560)
Supplement: Supplementary file 1 [file jcm-14-07560-s001.zip › Supplementary Material 2 - COSQ in english.pdf]

Chronic Obstructive Sialoadenitis quality-of-life Questionnaire (CSOQ) [Santillan et al, 2022]

Local symptoms

1. In the past three months, how much does the affected salivary gland hurt when you touch or press on it?

0	1	2	3	4
None	A little	Somewhat	Quite a lot	Very much

2. In the past three months, <u>during meals</u>, how often do you feel swelling of the affected gland or surrounding areas?

0	1	2	3	4
Never	Rarely	Sometimes	Very often	Always

3. In the past three months, <u>between meals</u>, how often do you feel swelling of the affected gland or surrounding areas?

0	1	2	3	4
Never	Rarely	Sometimes	Very often	Always

Functional symptoms

4. In the past three months, how often do you notice dry mouth (reduced amount of saliva)?

0	1	2	3	4
Never	Rarely	Sometimes	Very often	Always

5. In the past three months, does the swelling of the affected gland bother you when you open your mouth, yawn or chew?

0	1	2	3	4
None	A little	Somewhat	Quite a lot	Very much

6. In the past three months, does the swelling of the affected gland bother you when talking or swallowing?

0	1	2	3	4
None	A little	Somewhat	Quite a lot	Very much

Social affection

7. In the last three months, when the swelling occurs, do people around you notice it?

0	1	2	3	4
Never	Rarely	Sometimes	Very often	Always

8. In the past three months, are you ashamed to be seen in public when these symptoms occur?

0	1	2	3	4
Never	Rarely	Sometimes	Very often	Always

9. In the last three months, have you changed your eating habits (i.e avoiding any specific foods) because of your symptoms?

0	1	2	3	4
Not at all	A little	Somewhat	Quite a lot	Completely

10. In the past three months, have you changed your social or family habits?

0	1	2	3	4
Not at all	A little	Somewhat	Quite a lot	Completely

11. In the past three months, have your symptoms affected your sleep or night`s rest?

0	1	2	3	4
Never	Rarely	Sometimes	Very often	Always

Occupational impairment

12. In the last three months, do your symptoms affect your work, academic or recreational activities?

0	1	2	3	4
Never	A little	Somewhat	Quite a lot	Completely

13. In the last three months, due to salivary gland inflammation, have you been forced to miss any work, school and/or recreational activities?

0	1	2	3	4
Never	A little	Somewhat	Quite a lot	Completely

Emotional affection

14. In the past three months, does the swelling of your gland cause irritability or moodiness?

0	1	2	3	4
Never	Rarely	Sometimes	Very often	Always

15. In the past three months, does the swelling of your gland cause you to feel sad or discouraged?

0	1	2	3	4
Never	A little	Somewhat	Quite a lot	Completely

Global health impact

16. In the last three months, how many times have you needed to take painkillers or go to the emergency department because of your salivary gland complaints?

0	1	2	3	4
Never	Few<br>(≤ twice)	Sometimes<br>(3-4 times)	Quite often<br>(5-6 times)	Very often<br>(≥7 times)

17. In the past three months, how do you think the swelling of your salivary gland affects your health?

0	1	2	3	4
None	A little	Somewhat	Quite a lot	Very much

18. In the last three months, taking into account the swelling of your salivary gland, how do you rate your general health?

0	1	2	3	4
Excellent	Good	Normal	Poor	Very bad

Thanks for your cooperation.

Extracted from de Santillán Coello JM et al. Validation of a Spanish chronic obstructive sialadenitis quality of life questionnaire (CSOC). Acta Otorrinolaringol Esp (Engl Ed). 2023 Mar-Apr;74(2):116-123.
