# Supplementary material for: Quantifying the Impact of Chronic Obstructive Sialadenitis on Quality of Life
Source: J Clin Med. 2025 Oct 24;14(21):7560. doi: 10.3390/jcm14217560 (PMC12608179; doi:10.3390/jcm14217560)
Supplement: Supplementary file 1 [file jcm-14-07560-s001.zip › Supplementary Material 3.pdf]

Supplementary Material 3. Relationship between epidemiological factors, obstructive causes, and dilation. (C) Column percentages and (R) Row percentages.

	**Obstructive factor**												**Obstructive consequence**		
**Epidemiological factor**	**Stenosis**<br>(n=165)			**Lithiasis**<br>(n=111)			**LPD**<br>(n=68)			**Mucus plug**<br>(n=25)			**Dilatation**<br>(n=31)		
**Age of patient**<br>(median [range])	*NO*	*YES*	p	*NO*	*YES*	p	*NO*	*YES*	p	*NO*	*YES*	p	*NO*	*YES*	p
	51<br>[16-80]	53<br>[14-76]	0.19	53<br>[14-80]	49<br>[16-76]	**0.026**	50<br>[14-56]	53.5<br>[17-80]	**0.048**	52<br>[14-80]	48<br>[21-69]	**0.03**	52<br>[14-80]	54<br>[23-75]	**<0.005**
**Years of evolution**<br>(median [range])	*NO*	*YES*		*NO*	*YES*		*NO*	*YES*		*NO*	*YES*		*NO*	*YES*	
	2<br>[0.2-28]	3<br>[0.3-26]	0.15	3<br>[0.3-26]	2<br>[0.2-28]	**0.007**	2<br>[0.2-28]	3<br>[0.3-20]	0.178	2<br>[0.20-28]	5<br>[0.33-20]	**<0.005**	2<br>[0.2-28]	3<br>[0.33-20]	0.28
**Sex**	*NO*	*YES*	p	*NO*	*YES*	p	*NO*	*YES*	p	*NO*	*YES*	p	*NO*	*YES*	p
Male<br>(25,58%, n=88)	61<br>34.07% (C)<br>69.31% (R)	27<br>16.36% (C)<br>30.68% (R)		39<br>16.74% (C)<br>44.32% (R)	49<br>44.14% (C)<br>55.68% (R)		78<br>27.74% (C)<br>88.64% (R)	10<br>14.71% (C)<br>11.36% (R)		81<br>25.39% (C)<br>92.05% (R)	7<br>28% (C)<br>7.95% (R)		81<br>25.88% (C)<br>92.95% (R)	7<br>22.58% (C)<br>7.95% (R)	
Female<br>(74,43%, n=256)	118<br>65.92% (C)<br>46.09% (R)	138<br>83.63% (C)<br>53.90% (R)	**<0.005**<br>(OR 2.63)	194<br>83.26% (C)<br>75.78% (R)	62<br>55.86% (C)<br>24.22% (R)	**<0.005**<br>(OR 3.94)	198<br>72.26% (C)<br>77.34% (R)	58<br>85.29% (C)<br>22.66% (R)	**0.031**<br>(OR 2.28)	238<br>74.61% (C)<br>93.75% (R)	18<br>71% (C)<br>7.03% (R)	0.96	232<br>74.12% (C)<br>90.63% (R)	24<br>77.42% (C)<br>9.38% (R)	0.85
**Affected gland**	*NO*	*YES*	p	*NO*	*YES*	p	*NO*	*YES*	p	*NO*	*YES*	p	*NO*	*YES*	p
Parotid<br>(50%, n=172)	47<br>27.32% (C)<br>26.25% (R)	125<br>75.76% (C)<br>72.67% (R)		145<br>62.24% (C)<br>84.30% (R)	27<br>24.32% (C)<br>15.70% (R)		160<br>57.97% (C)<br>93.02% (R)	12<br>17.65% (C)<br>6.98% (R)		151<br>47.33% (C)<br>87.79% (R)	21<br>84% (C)<br>12.21% (R)		148<br>47.29% (C)<br>86.05% (R)	24<br>77.42% (C)<br>13.95% (R)	
Submandibular<br>(50%, n=172)	132<br>72.67% (C)<br>76.74% (R)	40<br>24.24% (C)<br>23.25% (R)	**<0.005**<br>(OR 8.7)	88<br>37.76% (C)<br>51.16% (R)	84<br>75.68% (C)<br>48.84% (R)	**<0.005**<br>(OR 5.12)	116<br>27.74% (C)<br>67.44% (R)	56<br>82.53% (C)<br>32.56% (R)	**<0.005**<br>(OR 6.43)	168<br>52.67% (C)<br>97.67% (R)	4<br>16% (C)<br>2.33% (R)	**<0.001**<br>(OR 5.84)	165<br>52.71% (C)<br>95.35% (R)	7<br>22.58% (C)<br>4.65% (R)	**<0.005**<br>(OR 3.75)
**Side**	*NO*	*YES*	p	*NO*	*YES*	p	*NO*	*YES*	p	NO	YES	p	*NO*	*YES*	p
Right<br>(50%, n=172)	94<br>52.51% (C)<br>54.65% (R)	78<br>47.27% (C)<br>45.35% (R)		113<br>48.30% (C)<br>65.70% (R)	59<br>53.15% (C)<br>34.30% (R)		138<br>50% (C)<br>80.23% (R)	34<br>50% (C)<br>19.76% (R)		160<br>50.15% (C)<br>93.02% (R)	12<br>48% (C)<br>6.97% (R)		152<br>48.55% (C)<br>88.37% (R)	20<br>64.52% (C)<br>11.63% (R)	
Left<br>(50%, n=172)	85<br>47.49% (C)<br>49.42% (R)	87<br>52.73% (C)<br>50.58% (R)	0.38	120<br>51.50% (C)<br>69.67% (R)	52<br>46.85% (C)<br>30.23% (R)	0.48	138<br>50% (C)<br>80.23% (R)	34<br>50% (C)<br>19.76% (R)	1	159<br>49.84% (C)<br>92.44% (R)	13<br>52% (C)<br>7.55% (R)	1	161<br>51.45% (C)<br>93.60% (R)	11<br>35.48% (C)<br>6.40% (R)	0.132
