# Supplementary material for: Quantifying the Impact of Chronic Obstructive Sialadenitis on Quality of Life
Source: J Clin Med. 2025 Oct 24;14(21):7560. doi: 10.3390/jcm14217560 (PMC12608179; doi:10.3390/jcm14217560)

Robustness of Gland-Level Analysis in the COSQ Study

1. Purpose

This supplementary document provides a statistical justification for analyzing the COSQ (Quality-of-Life questionnaire for Obstructive Sialadenitis) at the **gland level**, rather than aggregating at the **patient level**, by demonstrating that intra-patient dependence exists and is properly accounted for using appropriate statistical techniques.

2. Statistical Methods

- **Intra-patient correlation**: Assessed via the **Intraclass Correlation Coefficient (ICC[1,1])** using 300 bootstrap replicates.
- **Multilevel correction**: Mixed-effects linear model including a random intercept for patient (1 | Patient) to correct for the clustered structure.
- **Negative control**: Replication of main analyses in a subsample of **monoglandular patients**.
- **Criterion validity**: Association between COSQ scores and objective clinical severity markers at the gland level (stenosis grade, stone size).

3. Detailed Results

3.1 Intra-patient dependence

Supplementary Table 4.1. Intraclass Correlation Coefficient (ICC)

Metric	Value	95% CI	% variance explained by patient	Interpretation
ICC (1,1)	0.982	0.971–0.992	98%	Very high similarity between glands of the same patient ⇒ clustering must be corrected.

3.2 Mixed-effects vs. simple models

Supplementary Table 4.2. Fixed effects in mixed vs. simple models

Variable (ref.)	β ± SE (Mixed model)	p	β ± SE (Simple model)	p
Female vs. Male	+6.28 ± 1.82	0.001	+6.34 ± 1.73	<0.001
Stenosis vs. Lithiasis	+3.63 ± 1.71	0.034	+5.20 ± 2.04	0.011
LPD vs. Lithiasis	+3.57 ± 1.78	0.045	+5.94 ± 2.33	0.011
Mixed type vs. Lithiasis	+4.14 ± 1.95	0.034	+3.82 ± 2.54	0.134
Parotid vs. Submandibular	+1.84 ± 1.38	0.182	+4.58 ± 1.78	0.010

After adjusting for the patient effect ($\sigma^2 = 161.3$), estimates for sex and obstruction type remain consistent and significant; only the effect of gland location becomes non-significant, suggesting it was overestimated in the simpler model.

3.3 Negative control: monoglandular patients

Supplementary Table 4.3. Subanalysis in 215 patients with only one affected gland

Variable	β ± SE	p	Matches mixed model?
Female	+3.60 ± 1.94	0.065	Same direction; less power
Stenosis	+4.78 ± 2.36	0.044	Yes
LPD	+8.95 ± 2.78	0.001	Yes (even stronger)
Mixed type	+3.93 ± 2.84	0.167	Similar trend
Parotid	+0.37 ± 2.07	0.858	Same null effect

3.4 Criterion validity at the gland level (Supplementary Table 4.4)

Local severity marker	Spearman ρ	p	Interpretation
Stenosis grade (S1–S4)	+0.210	0.007	Higher stenosis = worse QoL
Stone size (mm)	–0.016	0.88	No correlation (clinically plausible)

3.5 Model assumptions

Supplementary Figure 4.1. Residual plot of the mixed model. The residuals are evenly distributed without systematic patterns or heteroscedasticity, supporting the adequacy of the model specification.

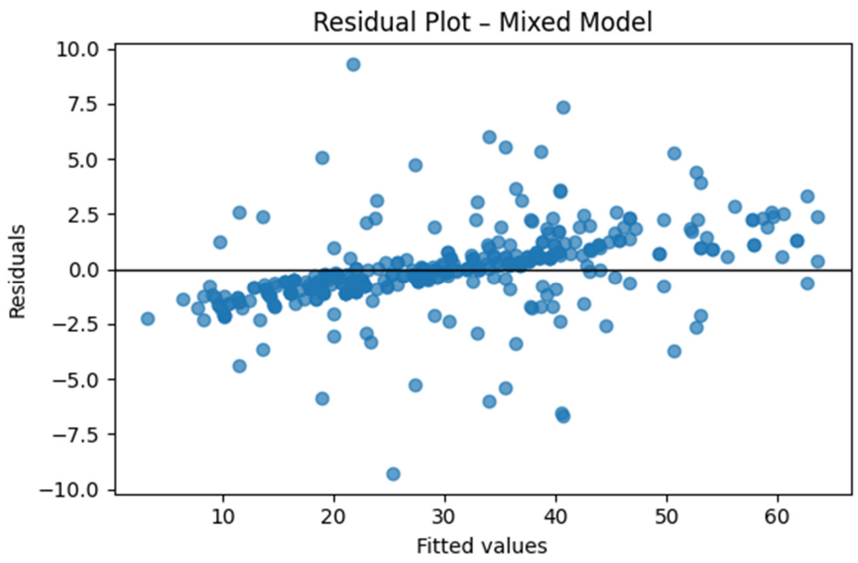

Supplement: Supplementary file 1 [file jcm-14-07560-s001.zip › Supplementary Material 4.pdf]
