# Supplementary material for: Quantifying the Impact of Chronic Obstructive Sialadenitis on Quality of Life
Source: J Clin Med. 2025 Oct 24;14(21):7560. doi: 10.3390/jcm14217560 (PMC12608179; doi:10.3390/jcm14217560)
Supplement: Supplementary file 1 [file jcm-14-07560-s001.zip › Supplementary Material 5.pdf]

Supplementary Material 5. Comparison of COSQ values (mean ± standard deviation) between single- and multi-gland involvement groups.

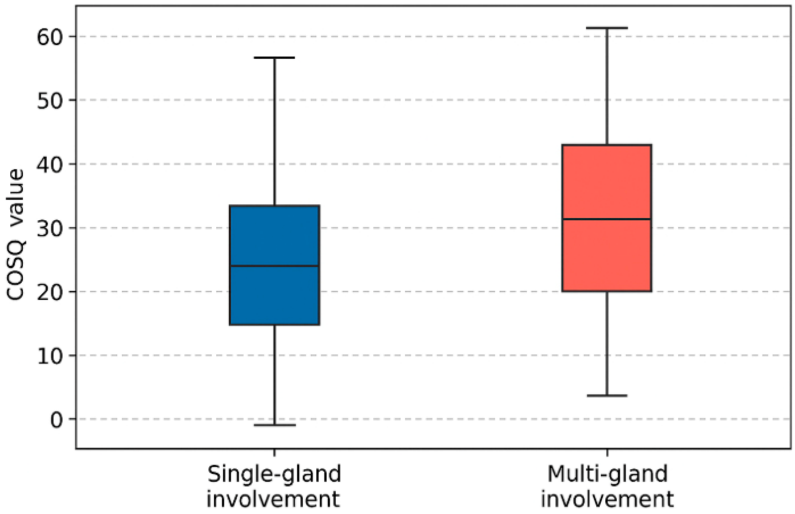

COSQ value	Single-gland involvement (n=212)	Multi-gland involvement (n=132)	p value
Median (±SD)	27.19 (±13.11)	35.95 (±14.38)	0.0001
